# Supplementary material for: Receiving support for mental health problems from family and friends: Measurement and impact on mental health, relationships, and help-seeking
Source: PLOS Ment Health. 2025 Dec 4;2(12):e0000502. doi: 10.1371/journal.pmen.0000502 (PMC12798267; doi:10.1371/journal.pmen.0000502)
Supplement: Attachment — Submitted filename: Response to reviewers final.docx [file pmen.0000502.s002.docx]

# Response to reviewers

**PMEN-D-25-00367: Receiving support for mental health problems from family and friends: Measurement and impact on mental health, relationships, and help-seeking**

Journal Requirements:

1. Please describe in your methods section how capacity to provide consent was determined for the participants in this study. Please also state whether your ethics committee or IRB approved this consent procedure. If you did not assess capacity to consent please briefly outline why this was not necessary in this case.

Response: This is on page 5, lines 128-129.

2. Please amend your detailed Financial Disclosure statement. This is published with the article. It must therefore be completed in full sentences and contain the exact wording you wish to be published.

i. State the initials, alongside each funding source, of each author to receive each grant.
ii. State what role the funders took in the study. If the funders had no role in your study, please state: “The funders had no role in study design, data collection and analysis, decision to publish, or preparation of the manuscript.”

Response: We have amended this, please see page 24.

3. Please send a completed 'Competing Interests' statement, including any COIs declared by your co-authors. If you have no competing interests to declare, please state "The authors have declared that no competing interests exist". Otherwise please declare all competing interests beginning with the statement "I have read the journal's policy and the authors of this manuscript have the following competing interests:"

Response: This has been provided in the manuscript (pg24) and in the online submission system.

4. Please ensure that your Ethics Statement is available in its entirety at the beginning of your Methods section, under a subheading 'Ethics Statement'.

Response: We have moved this to page 5, as requested.

5. In the online submission form, you indicated that “The data that support the findings of this study are available on request from the corresponding author, Amy Morgan.”.

All PLOS journals now require all data underlying the findings described in their manuscript to be freely available to other researchers, either 
1. In a public repository, 
2. Within the manuscript itself, or 
3. Uploaded as supplementary information.

This policy applies to all data except where public deposition would breach compliance with the protocol approved by your research ethics board. If your data cannot be made publicly available for ethical or legal reasons (e.g., public availability would compromise patient privacy), please explain your reasons by return email and your exemption request will be escalated to the editor for approval. Your exemption request will be handled independently and will not hold up the peer review process, but will need to be resolved should your manuscript be accepted for publication. One of the Editorial team will then be in touch if there are any issues.

Response: We have uploaded the data underlying the study to Figshare, <https://doi.org/10.26188/30561206.v1>. We will remove the embargo once the paper is accepted for publication.

If the reviewer comments include a recommendation to cite specific previously published works, please review and evaluate these publications to determine whether they are relevant and should be cited. There is no requirement to cite these works unless the editor has indicated otherwise. 

Please review your reference list to ensure that it is complete and correct. If you have cited papers that have been retracted, please include the rationale for doing so in the manuscript text, or remove these references and replace them with relevant current references. Any changes to the reference list should be mentioned in the rebuttal letter that accompanies your revised manuscript. If you need to cite a retracted article, indicate the article’s retracted status in the References list and also include a citation and full reference for the retraction notice.

Response: This has been done and there are no retracted papers cited.

5. Review Comments to the Author

**Reviewer #1:** This review was removed by journal staff as the comments provided were not relevant to this submission.

**Reviewer #2:** This manuscript presents a well-executed and timely investigation into the quality of informal mental health support and its impact on recipients. The research is methodologically sound, with a large sample size and robust psychometric validation, and contributes meaningfully to the literature on community-based mental health support.

Areas for Improvement
1) Recall Bias: The 12-month recall period may introduce memory inaccuracies; a shorter timeframe could improve reliability. Propably it has to be more clear indicated in the Limitation section

Response: We have edited the limitations section to more clearly articulate this issue, see page 22, line 385-386: “The 12-month recall period may have introduced memory inaccuracies and increased measurement error.”

2) MHSS-Received psychometrics analysis distract from the main article`s content. Maybe it is possible at least the tables to move to the Annexes. Otherwise, I would recommend to publish these data as a separate article.

Response: We are unsure which Tables the reviewer refers to, but assume they mean Table 2 and Table 3, which report the sample proportion who received each action, and the descriptive information for all outcome measures, respectively. We would prefer to keep these tables in the main text, for the following reasons: (1) We think these tables report major findings from the study so they should not be in supplementary files, (2) it would not be an efficient use of journal resources to publish two articles on these findings when it can be done in one, (3) the paper fits the length recommendations of the journal, and (4) Reviewer 3 said the results were well presented and the tables were clear.

**Reviewer #3:** Thanks for the opportunity to review this interesting study on Mental Health First Aid and the development of a questionnaire for people who have received MHFA to self report from their perspective of receiving help. This kind of measure contribute towards the evaluation of such programmes and training for the individual receiving it on what is a very difficult to measure interpersonal process - when someone will use the training and in what way and how long for can be unpredictable and so this design focuses on the recipient to retrospectively consider interactions that resulted in them feeling helped, which can theoretically confirm that skills taught on MHFA are helpful.

Introduction
The introduction effectively outlines the key concepts related to MHFA and is clear and well written. There majority of the discussion is on MHFA and little on the key dynamics of the people being studied. This section could benefit from increased discussion on the role of family and friends in mental health support and in the help-seeking process. This area is often assumed or briefly mentioned despite being very complex. In acknowledgement of this, expanding briefly/signposting to other research on how informal sources of help act as helpers, blockers, facilitators to help, support while getting help, maintenance, out of hours support etc. can deepen the understanding on why MHFA is so important for communities, with ripple effects into society on stigma, discrimination, or misunderstanding of mental health issues, and larger impacts on health/life outcomes. Expanding this discussion would both enhance the literature linkage and provide stronger justification for the research focus. This links with the discussion.

Response: We have expanded the discussion of the complex role that friends and family play in mental health support and the help-seeking process, including adding four new references to provide more context for the study. Please see revised text in the introduction page 3:

“The role of friends and family members in providing mental health support can be complex. Discrimination from family and friends is a common experience reported by people with mental health problems, such as people avoiding or excluding them or expressing judgemental and dismissive attitudes about their problem or the need for treatment.(2, 3) In contrast, friends and family members can also act as initial facilitators of help-seeking (4-6) by recognising emerging symptoms, deciding to intervene, and encouraging professional help, especially when it is considered appropriate to their relationship and they have the ability and capacity to help.(7) This is important because world-wide data show there remains a significant gap in the uptake of professional treatments for mental health problems (8) and there can be considerable delays before help is sought.(9, 10) Friends and family members can also be important sources of support during treatment and recovery, by providing emotional support, practical help with everyday tasks and navigating the health system, and encouraging engagement in treatment and self-care activities.(2, 11, 12) Improving the knowledge and mental health support skills of family members and friends is therefore one avenue to facilitating early intervention and improving population mental health.(13)”

Methodology
The description of participants, sampling procedures, and measures is clear and appropriately detailed. The adaptation of the MHSS to the (MHSS-Received) appears transparent and the use of a wide range of established measures for validity checking is thoroughly documented. One area that could be improved is further information on debriefing and/or aftercare. Being transparent about these processes is important for information sharing and best practice reporting. Including a brief discussion of how participants were supported post-study (e.g., debriefing procedures or access to resources) would demonstrate ethical rigor and good research practice.

Response: We have added a description of the debriefing procedures to the Method section on page 6-7: “Upon completion, participants were encouraged to contact their local doctor or mental health provider if the survey had brought up difficult emotions or they wanted mental health support and were also provided with a list of confidential support options they could access (e.g. Samaritans helpline in the UK).”

Results and discussion
The results are well-presented, confirming what is known or what is being taught on MHFA courses. There are clear tables and effective use of narrative summaries. The discussions on validity and interpretation are well-aligned with the data. The discussion makes a persuasive case for the benefits of mental health first aid training. However, it could go further in articulating why these findings matter beyond the immediate context. This links back with the comments in the introduction, authors could emphasize how upskilling community members in mental health first aid could contribute to broader public health outcomes -such as increasing early intervention, reducing stigma, and strengthening community-based support networks. Expanding on these implications would increase the practical relevance and societal value of the study.

Response: Thank you for this feedback. We have expanded the second paragraph of the Discussion to emphasise how upskilling community members could have public health benefits, see page 21 lines 351-353: “These findings underscore the important role of family and friends as community-based support networks who can contribute to increased early intervention, greater professional help-seeking, reduced stigma and discrimination and ultimately improved population mental health.” We have also strengthened the conclusion to further emphasise the implications of the findings, see page 24 lines 422-423: “Improving the mental health support provided within social networks could have a significant positive impact on population mental health.”
